# Screening participation after a false positive result in organized cervical cancer screening: a nationwide register-based cohort study

**DOI:** 10.1038/s41598-020-72279-x

**Published:** 2020-09-22

**Authors:** Pernille Thordal Larsen, Susanne Fogh Jørgensen, Mette Tranberg, Sisse Helle Njor

**Affiliations:** 1 Department of Public Health Programmes, Randers Regional Hospital, Skovlyvej 15, 8930 Randers NØ, Denmark; 2 Department of Clinical Medicine, Aarhus University, Palle Juul-Jensen Boulevard 82, 8200 Aarhus N, Denmark

**Keywords:** Cancer screening, Gynaecological cancer

## Abstract

Our aim was to investigate whether receiving a false positive (FP) cervical cytology result affected subsequent cervical cancer screening participation. This Danish nationwide register-based cohort study included 502,380 women aged 22.5–45 attending cervical cancer screening in 2012–2014 with a normal (n = 501,003) or FP (n = 1,377) cytology screening result. A FP result was defined as a cervical cytology showing high grade cytological abnormalities followed by a normal or ‘Cervical Intraepithelial Neoplasia grade 1’ biopsy result. Women were categorized as subsequent participants if they had a cervical cytology within 24–42 months after their last screening or surveillance test. We compared subsequent participation among women with a normal versus a FP result, using odds ratios including 95% confidence intervals. Participation was slightly higher among women with FP results than among women with normal results (71.5% vs. 69.2%, p = 0.058). After adjustment for age and screening history, women with FP results participated significantly more than women with normal results (OR: 1.19, 95% CI 1.06–1.35). Women receiving a FP result did not participate less in subsequent cervical cancer screening than women receiving a normal result. In fact, the use of opportunistic screening seemed to be increased among women receiving a FP result.

## Introduction

The introduction of organized screening programs has contributed to a decline in cervical cancer incidence and mortality in several Western countries^1–3^. The preventive effect of cervical cancer screening programs however depends on high participation^4^. As number of false positive tests are expected to increase with the introduction of human papillomavirus (HPV) based cervical cancer screening^5^, it is important to know if receiving a false positive test affects subsequent cervical cancer screening participation, as this will then reduce the preventive effect of HPV based cervical cancer screening.

Questionnaire studies have reported that receiving an abnormal result in cervical cancer screening gives rise to anxiety and reduced quality of life^6–9^. Even though anxiety fades during follow-up^6–9^, an abnormal result is still perceived as an important event by the women several years later^7^. To our knowledge, no previous studies have evaluated whether receiving a false positive test affects subsequent cervical cancer screening participation.

This study aimed at investigating whether receiving a false positive screening result within an organized cervical cancer screening program is associated with subsequent cervical cancer screening participation. As most women with low-grade cytological abnormalities does not get a subsequent histology it would be hard to know whether they are truly false positive, we therefore chose only to include false positives among women with high-grade cytological abnormalities.

## Materials and methods

### Setting

Organized cervical cancer screening was introduced in some Danish counties in the 1960s. By the late 1990s, 90% of Danish women were covered by the screening program, while nationwide coverage was achieved for women aged 23–59 in 2006^10^. The organization of the screening program is defined nationally, but daily operation is managed individually in each of the five Danish regions.

Women aged 23–49 years are invited for cervical cancer screening every third year, while women aged 50–64 years are invited for screening every fifth year^10^. This results in approximately 1.5 million women in the Danish target population for cervical cancer screening^11^. Women are invited to book a screening appointment with their general practitioner (GP) when the age-specific interval has passed since their latest invitation or cervical cytology sample (whichever came last)^12^. In case of non-participation, reminders are sent at 3 and 6 months after the initial invitation. The primary screening method for 23–59-year-old women are microscopic examination of the cytology sample, while 60–64-year-old women are offered a human papillomavirus (HPV) DNA check-out test^12^. Cytology are categorized per the Bethesda 2001 classification as: normal, inadequate, Atypical Squamous Cells of Undetermined Significance (ASC-US), Low-grade Squamous Intraepithelial Lesion (LSIL), Atypical Squamous Cells cannot exclude HSIL (ASC-H), High-grade Squamous Intraepithelial Lesion (HSIL), Squamous Cell Carcinoma (SCC), Atypical Glandular Cells (AGC), Adenocarcinoma In Situ (AIS), Adenocarcinoma (ACC), and malignant tumor cells^13^. Outside the organized screening program, GPs or gynecologists can obtain opportunistic cervical samples, also in women below 23 years. The screening program as well as any opportunistic testing, surveillance or treatment is free of charge^14^.

**Figure 1:**
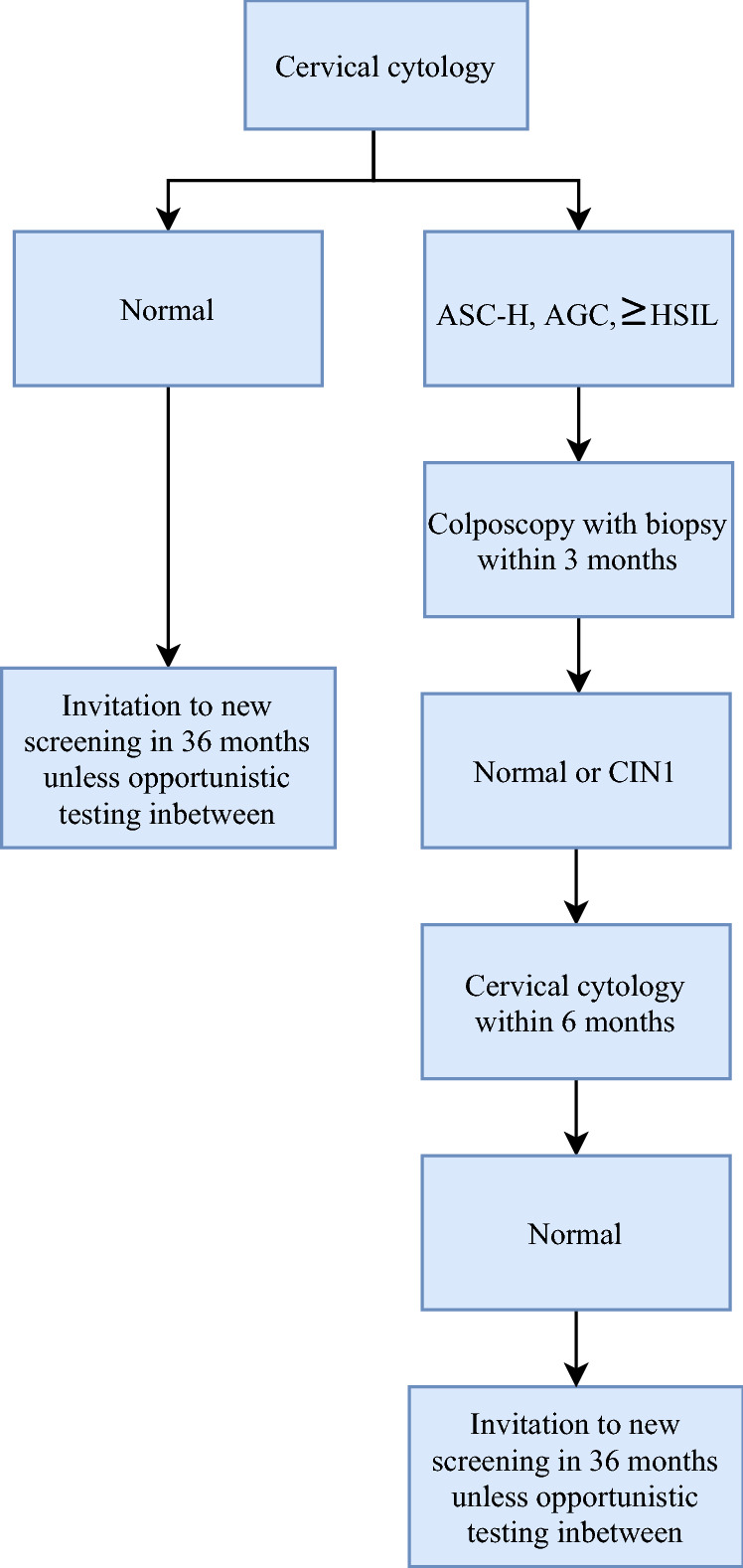
Flowchart of recommendations for the normal and false positive group. *ASC-H* atypical squamous cells cannot exclude HSIL, *AGC* atypical glandular cells, *HSIL(ASC-H)* ≥ HSIL: atypical squamous cells cannot exclude, *HSIL* high-grade squamousintraepithelial lesion, *SCC* squamous cell carcinoma, *AIS* adenocarcinoma in situ, *ACC* adenocarcinoma, *malignant tumor cells CIN1* cervical intraepithelial neoplasi grade 1.

If a cervical cytology sample is inadequate or abnormal (≥ ASC-US), the woman is enrolled in a surveillance program. For women with low-grade cytological abnormalities (ASC-US and LSIL), this includes either triage with HPV reflex testing or repeat cytology testing^12^. Women with high-grade cytological abnormalities (ASC-H, AGC, HSIL+) are referred directly to colposcopy for cervical biopsy (a surveillance biopsy) within 3 months^15^ (Fig. 1). Cervical biopsies are graded using the Cervical Intraepithelial Neoplasia (CIN) classification as: normal (including inflammation and non-specific reactive features), CIN grade 1, 2 or 3/AIS, or invasive cancer^16^. If the surveillance biopsy is normal or shows CIN1, the woman is recommended a second cervical cytology sample after 6 months. The woman returns to the screening program if no abnormalities are detected at this second surveillance examination^15^ (Fig. 1).

### Study design and study population

This study is a nationwide register-based cohort study. Women without a previous cervical cancer diagnosis aged 22.5–45 years were included if they were registered with a cervical cytology screening sample (index test) with either a normal screening result or a false positive result of high-grade cytological abnormalities (ASC-H, AGC, HSIL+) in the Danish National Pathology Registry (DNPR) between 1 January 2012 and 31 December 2014. These women were followed until next cervical cancer screening sample (index test) or 31 December 2019, which ever came first.

Women were excluded if they during the follow-up period: died or emigrated from Denmark, had an abnormal cervical cytology or biopsy (> CIN1), had a conization or hysterectomy performed, or if they never had 24 months in between tests, as this would make it unclear whether they had prolonged surveillance or frequent testing.

### Data sources

All residents in Denmark are registered in the Danish Civil Registration System with a unique ten-digit civil register number (CRN)^17^.The CRN allowed us to link individual-level data from four Danish registers: the Danish Civil Registration System, DNPR^18^, The Danish Cancer Register^19^, and the National Patient Register^20^.

Since 1997, all analyzed pathology specimen in Denmark have been registered in DNPR. The DNPR uses the Danish version of the Systematized Nomenclature of Medicine (SNOMED) to store detailed pathology information^18^. We identified all cervical cytologies, biopsies, and conizations in the DNPR using the SNOMED codes: T8X2*, T8X3*, T82000 and T83*. For each sample, we identified date and diagnosis using the SNOMED codes based on the Bethesda classification.

In the Danish Cancer Register, we identified the date of any cervical cancer diagnoses (ICD-10: DC53*, ICD-7: 171*, 4710, 7710, 8710^21^); and in the National Patient Register, we identified the date of any hysterectomy (ICD-10: KLCD*, KLDC10*, KLDC13, KLDC20, KLDC23, KLDC96, KLEF00B, KLEF13, KMCA33^22^). The Danish Civil Registration system gave information on date of birth, migration, death or any other reason for CRN inactivation^17^.

### Definitions of index test and a false positive screening result

The index test was defined as the first cervical cytology sample in the study period carrying a SNOMED code for a screening cytology (T8X2*, T8X3*). To verify that the index test was a screening sample, it was a requirement that the there was no cytological or histological abnormalities, surveillance code, or unsubscription code in the preceding 24 months prior to the index test, as this could indicate that it was not a screening test.

Based on the results of the index test, women were allocated into two groups: (1) women with a normal screening result and (2) women with a false positive screening result. A false positive screening result was defined as an index test classified as high-grade cytological abnormalities (ASC-H, AGC, HSIL+) with a subsequent ≤ CIN1 cervical biopsy result within the next 6 months.

Participation in the subsequent screening round was defined as a record of a cervical cytology sample (SNOMED:T8X2*-T8X3*) registered 24–42 months after the date of the index test or surveillance test. The 24-month margin was chosen to avoid cervical cytology samples that could be performed as part of surveillance or obtained opportunistically due to any symptoms.

### Confounding variables

A priori, we decided to check for three possible confounders. These included age at index test^23^, history of any abnormal cervical cytology or biopsy within the past 10 years, and participation in the last screening round prior to the index test^8,24^. Participation in the previous screening round was defined as having had a cervical cytology within 42 months prior to the index test.

### Statistics

Data on age, screening history and conizations in the two groups are presented in numbers and percentages. The distribution of these variables between the groups was compared using chi-square test. We compared participation in the subsequent screening round among women with a normal screening result and women with a false positive screening result. Using logistic regression we estimated and presented crude and adjusted OR, using women with a normal result as the reference group. We adjusted for age (continuous), history of abnormal cervical cytology or biopsy in the past 10 years (dichotomous: yes/no), and screening participation less than 42 months before the index test (dichotomous: yes/no).

We compared the proportion of participants who used opportunistic testing (< 36 months since last test) in subsequent screening among women with a normal result and women with a false positive result at the index test.

All estimates were presented with 95% confidence intervals (CI). All analyses were conducted using STATA statistical software version 15.1 (StataCorp., College Station, TX, USA).

### Ethics

According to the EU’s General Data Protection Regulation (article 30), the project was listed in the record of processing activities for research projects in the Central Denmark Region (J. No.: 1-16-02-301-18). According to the Consolidation Act on Research Ethics Review of Health Research Projects, Consolidation Act number 1083 of 15 September 2017 section 14 (2) notification of registry-based studies is only required if the project involves human biological material. Therefore, this study may be conducted without an approval from the Ethics Committees.

## Results

### Study population and baseline characteristics

**Figure 2:**
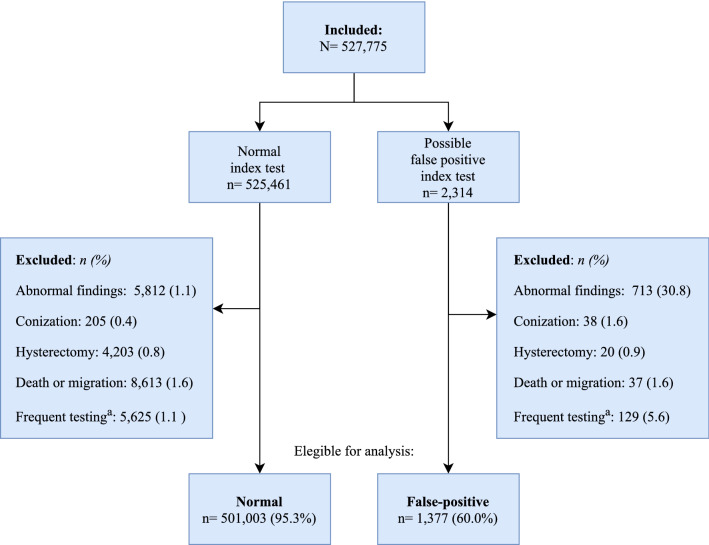
Flowchart of inclusion and exclusion. ^a^Women who never have 24 months in between tests during follow-up despite of no abnormal findings.

Among the 527,775 women included for follow-up, 525,461 had a normal index test, while 2,314 had a possible false positive test (Fig. 2). A total of 24,458 (4.7%) were excluded from the normal group, while 937 (40.5%) were excluded from the false positive group—primarily due to abnormal cytological or histological findings during follow-up (Fig. 2), i.e. these women did not after all have a false positive test.

Table 1 presents age and screening history stratified by the result of the index test. Compared to women with a normal index test, women with a false positive result were significantly less likely to have participated in cervical cancer screening within the past 42 months prior to the index test (57.9% vs. 61.9%, p = 0.002), but significantly more likely to have had an abnormal cervical test in the past 10 years (18.2% vs. 13.6%, p < 0.001). Women with a false positive result were younger (p < 0.001) than women with a normal result (Table 1).

**Table 1:** Distribution of baseline data among normal and false positive participants at inclusion.

N = 502,380	Normal		False positive		p-value
	*n* = 501,003		*n* = 1,377		
	*N*	%	*n*	%	*χ*^2^
**Age (years)**					
22.5–29	151,263	30.2	542	39.4	
29–34	102,575	20.5	281	20.4	
35–39	121,911	24.3	271	19.7	
40–45	125,254	25.0	283	20.6	< 0.001
**Screening history**					
Participation prior to index test^a^	310,084	61.9	797	57.9	0.002
Abnormality past 10 years^b^	68,313	13.6	251	18.2	< 0.001

Normal: defined as having an adequate cervical cytology result showing no dysplasia. False positive: defined as having an abnormal cervical cytology result showing high-grade cytological abnormalities with a subsequent biopsy with a normal or ‘cervical intraepithelial neoplasia grade 1’ result within 6 months.

^a^Cervical cytology performed within 42 months prior to inclusion.

^b^Any abnormal cervical results within the past 10 years prior to inclusion.

### Odds ratios of screening participation

Among women with a normal index test, 69.2% (95% CI 69.0%; 69.3%) participated in the subsequent screening round (n = 346,567) while 71.5% (95% CI 69.1%; 73.9%) of women with a false positive test participated in the subsequent screening round (n = 985). This resulted in a non-significantly increased crude OR for participation in the false positive group OR 1.12 (95% CI 1.00; 1.26) (Table 2). After adjustment for age and screening history, the association became stronger and reached statistical significance with OR 1.19 (95% CI 1.06; 1.35). No confounders interacted with the result of the index test or with age category. There were multiple interactions in between confounders, though none influenced the association in focus.

**Table 2:** Odds ratios of participation^a^ in subsequent screening after receiving a false positive cervical cytology screening (index test) result compared to a normal result.

N = 502,380	Normal^b^	False positive^c^	p-value
	n = 501,003	n = 1,377	
Participants, n	346,567	985	
% (95% CI)	69.2 (69.1; 69.3)	71.5 (69.1; 73.9)	
OR_crude_ (95% CI)	1 (ref.)	1.12 (1.00; 1.26)	0.059
OR_adjusted_^d^ (95% CI)	1 (ref.)	1.19 (1.06; 1.35)	0.004
Opportunistically screened^e^, n	71,217	408	
% (95% CI)	20.5 (20.4; 20.7)	41.4 (38.3; 44.5)	< 0.001

^a^Participation 24–42 months after the date of the index test or surveillance test:

^b^Adequate index test showing no dysplasia.

^c^Abnormal index test showing high-grade cytological abnormalities with a subsequent cervical biopsy with normal or ‘Cervical intraepithelial neoplasia grade 1’ result within 6 months.

^d^Adjusted for age, history of abnormal cervical test, and participation in screening within 42 months prior to the index test.

^e^Women who had a subsequent cervical cytology cancer screening 24 to < 36 months after the index test or cervical surveillance test.

Of the 985 women in the false positive group who participated in subsequent screening, 408 (41.4%) participated < 36 months after their last test. The same was true for 71,217 (20.5%) of the 346,567 women in the negative group who participated in subsequent screening, resulting in a statistically significant difference in use of subsequent opportunistic screening prior to invitation (Table 2).

## Discussion

In this register-based nationwide cohort study, we found that women with a false positive cervical cytology screening result were significantly more likely to participate in the subsequent screening round and more likely to use subsequent opportunistic screening than women with a normal screening result. To our knowledge, this is the first study that evaluates whether receiving a false positive test affects subsequent cervical cancer screening participation.

The use of high-quality register-based data instead of self-reported data is a key strength of this study as earlier studies found self-reported screening participation to be overestimated^25,26^. The proportion of missing data in the DNPR is considered extremely low, as information is obtained directly from the daily routine diagnostic tool used by all pathologists in Denmark^18^. This reduced the risk of selection and information bias and enhanced the internal validity of the study.

Defining a false positive result within cervical cancer screening can be challenging, as cervical dysplasia is a precursor of cervical cancer but does not necessarily develop into cancer^27^. Therefore, some of the abnormal cytology index tests categorized as false positives has been true positives that regressed prior to the cervical biopsy. False positive or not, this could not be known by the women and therefore had no effected on the results. In this study, we only looked at false positive tests arising from tests classified as high-grade cytological abnormalities, although there are also false positive tests arising from tests classified as low-grade cytological abnormalities. We do not know whether women with these kind of false positive tests participate more or less than women with a normal screening result.

We have no information on the indication for sampling, wherefore we do not know whether a cytology index test was collected as a screening sample, a surveillance sample, or a sample taken due to symptoms or on the woman's own initiative. By defining a screening index test based on no abnormal findings 24 months priori this test, we believe that we minimized the misclassification of the index test as much as possible. Unfortunately, having the same 24 months margin until subsequent screening meant that women could be excluded due to frequent opportunistic testing perceived as long surveillance. This is indicated by a larger proportion of women being excluded due to never having 24 months in between tests in the false positive group compared to the normal group (Fig. 2). In a sensitivity analysis we allowed next screening to be ≥ 9 months after last screening or surveillance tests. By this definition we could only include women who had both first and second surveillance tests within the recommended time-periods (+ 3 months), which undoubtedly meant that the included women in the false positive group in general were more compliant to the screening and surveillance recommendations. The association of a false positive result and subsequent participation was thus not surprisingly stronger with an adjusted OR of 2.00 (95% CI 1.52; 2.63) (Supplementary Table S1). While this is expected to be an overestimation of the true association, this indicates that the results of our primary analysis are probably underestimated.

As reminders are sent to women 6 months after their first invitation, women participating after this reminder are not counted as participants. We therefore made a sensitivity analysis were participation included cervical samples registered 24–48 months after the date of the index test/surveillance test, this resulted in a less strong, though still statistical significant adjusted OR of 1.15 (95% CI 1.001; 1.336) (Supplementary Table S2).

The youngest women did not have the opportunity of a previous screening. However, excluding women below 27 years did not change the results. As participation might not have a linear relationship with age, we also made all adjustments using age as a categorical variable. This did not change any of the results.

We had no information on socio-economic factors, pregnancies, and family history of cancer. Cervical cancer screening is not recommended during pregnancy^12^, which may explain some non-participation. We have no reason, though, to assume that pregnancy should be associated with false positive results and no major confounding is expected on this behalf. Hysterectomy data was unavailable after November 2018. This could potentially mean that some women should have been excluded, but was not. It is highly unlikely that this should have affected the results essentially as they would most likely be excluded due to abnormal findings anyway.

The higher screening participation and use of opportunistic screening among women with a previous false positive result than among women with a normal result is in line with the results of a qualitative review and meta-synthesis showing that false positive results in breast cancer screening seemed to create a desire and need for more screening^28^.

In several organized cervical cancer screening programs, HPV-based screening instead of cytology-based screening has already been implemented or is being considered for women aged 30 or older^10,29^. As HPV testing is more sensitive at detecting CIN2+, but less specific than cytology-based screening, the proportion of false positive results is expected to increase^5^. Our study shows that this will probably not affect screening participation and thereby not lower the effect of the screening programs. However, our study only included women below 45 years wherefore it is unknown if older women receiving a false positive result will also not have a reduced participation rate in subsequent cervical cancer screening.

Our findings also suggests that an increase in false positive result rates might result in more opportunistic screening, leading to reduced average screening intervals. This might be problematic, as a Danish study indicates that frequent opportunistic screening has no screen-related benefit^30^.

These results may be generalized to other countries with a similar organization of the cervical cancer screening program and free-of-charge healthcare systems.

In conclusion, women below 45 years receiving a false positive result do not participate less in subsequent cervical cancer screening than women receiving a normal result. The use of opportunistic screening seems to be increased among women receiving a false positive result.

## Supplementary information

Supplementary Tables.

## Data Availability

The data that support the findings of this study are available from The Danish Health Data Authority. Restrictions apply to the availability of these data, which were used under license for this study. Data may be available upon reasonable request to The Danish Health Data Authority.
