# Supplementary material for: Screening participation after a false positive result in organized cervical cancer screening: a nationwide register-based cohort study
Source: Sci Rep. 2020 Sep 22;10:15427. doi: 10.1038/s41598-020-72279-x (PMC7508879; doi:10.1038/s41598-020-72279-x)
Supplement: Supplementary file 1 — Supplementary Tables. [file 41598_2020_72279_MOESM1_ESM.docx]

Supplementary information

Screening participation after a false positive result in organized cervical cancer screening: A nationwide register-based cohort study

Pernille Thordal Larsen^1^, Susanne Fogh Jørgensen^1,2^, Mette Tranberg^1^, Sisse Helle Njor^1, 2*^

^1^Department of Public Health Programmes, Randers Regional Hospital, Skovlyvej 15, 8930 Randers NØ, Denmark, ^2^Department of Clinical Medicine, Aarhus University, Palle Juul-Jensen Boulevard 82, 8200 Aarhus N, Denmark

Supplementary Table S1

| *Table S1:* Odds ratios of participation in subsequent screening after receiving a false-positive cervical cytology screening result (index test), including early opportunistic screening | | | |
| --- | --- | --- | --- |
| *N=495,036* | Normal^a^  *n=494,670* | False-positive^b^  *n=366* | p-value |
| Participants ^c^, *n*  *% (95% CI)* | 353,953  71.6 (71.4;71.7) | 304  83.1 (78.8;86.8) |  |
| OR _crude_ (95% CI) | 1 (ref) | 1.95 (1.48;2.56) | <0.001 |
| OR _adjusted_ (95% CI) ^d^ | 1 (ref) | 2.52 (2.50;2.53) | <0.001 |

^a^ Defined as having an adequate cervical cytology result showing no dysplasia.

^b^ Defined as having an abnormal cervical cytology result showing high-grade cytological abnormalities with a subsequent biopsy with a normal or ‘cervical intraepithelial neoplasia grade 1’ result within 6 months and a subsequent negative cervical cytology within 3-9 months.

^c^ Women who had a cervical cytology 9 to 42 month after the index test or surveillance test.

^d^ Adjusted for age and history of abnormal cervical test within the past 10 years

Supplementary Table S2

| *Table S2:* Odds ratios of participation in subsequent screening after receiving a false-positive cervical cytology screening result (index test) allowing for four years screening interval. | | | |
| --- | --- | --- | --- |
| *N=500,973* | Normal^a^  *n=499,626* | False-positive^b^  *n=1,347* | p-value |
| Participants ^c^, *n*  *% (95% CI)* | 404,651  81.0 (80.9;81.1) | 1,106  82.1 (80.0;84.1) |  |
| OR _crude_ (95% CI) | 1 (ref) | 1.08 (0.94;1.24) | 0.297 |
| OR _adjusted_ (95% CI) ^d^ | 1 (ref) | 1.16 (1.00;1.34) | 0.048 |

^a^ Defined as having an adequate cervical cytology result showing no dysplasia.

^b^ Defined as having an abnormal cervical cytology result showing high-grade cytological abnormalities with a subsequent biopsy with a normal or ‘cervical intraepithelial neoplasia grade 1’ result within 6 months.

^c^ Women who had a cervical cytology 24 to 48 month after the index test or surveillance test.

^d^ Adjusted for age, history of abnormal cervical test, and participation in screening within 42 months prior to the index test
